# Supplementary material for: Quality of Information on Medication Abortion in Private Pharmacies: Results from a Mystery Client Study in Kinshasa, Democratic Republic of Congo
Source: Healthcare (Basel). 2025 Feb 24;13(5):491. doi: 10.3390/healthcare13050491 (PMC11899032; doi:10.3390/healthcare13050491)
Supplement: Supplementary file 1 [file healthcare-13-00491-s001.zip › Supplemental/supplementary material_ The scripts used during the visits (tranlated in english).pdf]

Mystery client Visit Script

Script 1: The Woman herself

- Walk up to the pharmacy counter and greet the person available to serve you.
- Explain your problem calmly (in Lingala)
  "Hello Sir/Madam, I think I have a problem because I haven't had my period for 9 weeks and I took a pregnancy test which came back positive and now I'm pregnant. I can't have a child right now and I wanted to know if there's anything I can do. Can you help me?"
- If the pharmacist asks you why, answer by **"I just had a cesarean delivery a year ago"**
- Pause at this point to see if the person makes any comments or asks you any questions.
- Remember that your answers should fit your character's story.
- If the person tells you that there is nothing they can do to help you, ask them why
- Ask them if they know of anyone else or somewhere else where someone could help you.
- **If they spontaneously ask you for a prescription: show them the prescription.**

Script 2: The mother

- Walk up to the pharmacy counter and greet the person available to serve you.
- Explain your problem calmly (in Lingala)
  "Hello Sir/Madam, I think I have a problem because my daughter hasn't had her period for 9 weeks and I did a pregnancy test which came back positive and now she is pregnant. She can't keep this pregnancy and I wanted to know if there is anything I can do. Can you help me?"
- If the pharmacist asks you why: answer by **"because there is a problem in the family, it is his cousin (my older sister's child) who is the author"**
- Pause at this point to see if the person makes any comments or asks you any questions.
- Remember that your answers must correspond to your character's story.
- If the person answers that they can't do anything to help you, ask them why.
- Ask them if they know of anyone else or somewhere else where someone could help you.
- **If they spontaneously ask you for a prescription: show the prescription.**

Script 3: The partner

- Walk up to the pharmacy counter and greet the person available to serve you.
- Explain your problem calmly (in Lingala)
  “Hello Sir/Madam, I think I have a problem because my wife hasn't had her period for 9 weeks and I took a pregnancy test which came back positive and now she is pregnant. We can't have a child right now and I wanted to know if there is anything I can do. Can you help me?”
- If the pharmacist asks you why: answer by **“she had a cesarean delivery just a year ago”**
- Pause at this point to see if the person makes any comments or asks you any questions.
- Remember that your answers should fit your character's story.
- If the person tells you that there is nothing they can do to help you, ask them why.
- Ask them if they know of anyone else or somewhere else that could help you.
- **If they spontaneously ask you for a prescription: show them the prescription.**

For all script: the woman herself, the mother or the partner

- If the person tells you to take a medicine (whether it’s Misoprostol or Mifepristone or something else), wait to see if they volunteer additional information.
- If not, ask them the following questions:
  - How should I take this medicine?
  - How many tablets at a time?
  - Do I take it with anything?
  - When should I take it? How long can I wait?
  - Does this medicine have any side effects?
  - Is it dangerous for my health?
  - Can I come back to see you if I have a problem?
  - Is there anything else I can do to prevent this pregnancy or getting my period again?

Note for all mystery client:

- Always ask the question innocently and as someone who is just trying to understand better (**even if you know the right answer, do not correct the person!**)
- We are trying to understand what kind of service a woman with little or no education would receive in this situation.
