# Supplementary material for: Quality of Information on Medication Abortion in Private Pharmacies: Results from a Mystery Client Study in Kinshasa, Democratic Republic of Congo
Source: Healthcare (Basel). 2025 Feb 24;13(5):491. doi: 10.3390/healthcare13050491 (PMC11899032; doi:10.3390/healthcare13050491)
Supplement: Supplementary file 1 [file healthcare-13-00491-s001.zip › Supplemental/supplementary material__ The scripts used during the visits in French.pdf]

Scenario des visites des clients mystères

Scenario 1 : La femme elle-même

- Avancez jusqu'au comptoir de la pharmacie et saluez la personne disponible pour vous servir.
- Expliquez tranquillement votre problème (en lingala)
  « Bonjour Monsieur/Madame, je pense que j'ai un problème parce que je n'ai pas eu mes règles depuis 9 semaines et j'ai fait un test de grossesse qui s'est avéré positif et maintenant je suis enceinte. Je ne peux pas avoir d'enfant pour l'instant et je voulais savoir si je pouvais faire quelque chose. Pouvez-vous m'aider ? »
- Si le pharmacien vous demande pourquoi : répondez par **« je venais d'un accouchement par césarienne il y a juste une année »**
- Marquez une pause à ce stade pour voir si la personne fait des commentaires ou vous pose des questions.
- N'oubliez pas que vos réponses doivent correspondre à l'histoire de votre personnage.
- Si la personne vous répond qu'elle ne peut rien faire pour vous aider, demandez-lui pourquoi
- Demandez-lui si elle connaît une autre personne ou un autre endroit où quelqu'un pourrait vous aider.
- **S'il vous demande spontanément une ordonnance : exhiber l'ordonnance.**

Scenario 2: La mère

- Avancez jusqu'au comptoir de la pharmacie et saluez la personne disponible pour vous servir.
- Expliquez tranquillement votre problème (en lingala)
  « Bonjour Monsieur/Madame, je pense que j'ai un problème parce que ma fille n'a pas eu ses règles depuis 9 semaines et j'ai fait un test de grossesse qui s'est avéré positif et maintenant elle est enceinte. Elle ne peux garder cette grossesse et je voulais savoir si je pouvais faire quelque chose. Pouvez-vous m'aider ? »
  Si le pharmacien vous demande pourquoi : répondez par « **car il y a un problème en famille, c'est son cousin (l'enfant à ma grande sœur) qui en est l'auteur** »
- Marquez une pause à ce stade pour voir si la personne fait des commentaires ou vous pose des questions.
- N'oubliez pas que vos réponses doivent correspondre à l'histoire de votre personnage.
- Si la personne vous répond qu'elle ne peut rien faire pour vous aider, demandez-lui pourquoi
- Demandez-lui si elle connaît une autre personne ou un autre endroit où quelqu'un pourrait vous aider.
- **S'il vous demande spontanément une ordonnance : exhiber l'ordonnance.**

Scenario 3: Le partenaire masculin

- Avancez jusqu'au comptoir de la pharmacie et saluez la personne disponible pour vous servir.
- Expliquez tranquillement votre problème (en lingala)
  « Bonjour Monsieur/Madame, je pense que j'ai un problème parce que ma femme n'a pas eu ses règles depuis 9 semaines et j'ai fait un test de grossesse qui s'est avéré positif et maintenant elle est enceinte. Nous ne pouvons pas avoir d'enfant pour l'instant et je voulais savoir si je pouvais faire quelque chose. Pouvez-vous m'aider ? »
- Si le pharmacien vous demande pourquoi : répondez par **« elle venait d'un accouchement par césarienne il y a juste une année »**
- Marquez une pause à ce stade pour voir si la personne fait des commentaires ou vous pose des questions.
- N'oubliez pas que vos réponses doivent correspondre à l'histoire de votre personnage.
- Si la personne vous répond qu'elle ne peut rien faire pour vous aider, demandez-lui pourquoi
- Demandez-lui si elle connaît une autre personne ou un autre endroit où quelqu'un pourrait vous aider.
- **S'il vous demande spontanément une ordonnance : exhiber l'ordonnance.**

Pour tous les scénarii : la femme elle-même, la mère ou le partenaire masculin

- Si la personne vous dit de prendre un médicament (qu'il s'agisse de Misoprostol ou de Mifépristone ou d'autre chose), attendez de voir si elle fournit spontanément des informations supplémentaires.
- Si ce n'est pas le cas, posez-lui les questions suivantes :
  - Comment dois-je prendre ce médicament ?
  - Combien de comprimés à la fois ?
  - Est-ce que je le prends avec quelque chose ?
  - Quand dois-je le prendre ? Combien de temps puis-je attendre ?
  - Ce médicament a-t-il des effets secondaires ?
  - Est-il dangereux pour ma santé ?
  - Puis-je revenir vous voir si j'ai un problème ?
  - Y a-t-il autre chose que je puisse faire pour éviter de garder cette grossesse ou d'avoir à nouveau mes règles ?

Note pour tous les clients mystères

- Posez toujours la question de manière innocente / comme quelqu'un qui cherche seulement à mieux comprendre (même si vous connaissez la bonne réponse, ne corrigez pas la personne !)
- Nous essayons de comprendre quel type de service une femme peu ou pas éduquée recevrait dans cette situation.
